# Leveraging an Implementation Science Framework to Measure the Impact of Efforts to Scale Out a *Total Worker Health*^®^ Intervention to Employers

**DOI:** 10.3390/ijerph19031372

**Published:** 2022-01-26

**Authors:** Liliana Tenney, Amy G. Huebschmann, Carol E. Brown, Natalie V. Schwatka, Lee S. Newman

**Affiliations:** 1 Center for Health, Work & Environment, Colorado School of Public Health, University of Colorado, Anschutz Medical Campus, 13001 E. 17th Pl., 3rd Floor, Mail Stop B119, Aurora, CO 80045, USA; carol.brown@cuanschutz.edu (C.E.B.); natalie.schwatka@cuanschutz.edu (N.V.S.); lee.newman@cuanschutz.edu (L.S.N.); 2 Department of Environmental and Occupational Health, Colorado School of Public Health, University of Colorado, Anschutz Medical Campus, 13001 E. 17th Pl., 3rd Floor, Mail Stop B119, Aurora, CO 80045, USA; 3 Adult & Child Consortium for Outcomes Research and Delivery Science (ACCORDS) Dissemination and Implementation Program, University of Colorado School of Medicine, Aurora, CO 80045, USA; amy.huebschmann@cuanschutz.edu; 4 Division of General Internal Medicine and Ludeman Family Center for Women’s Health Research, Anschutz Medical Campus, Aurora, CO 80045, USA; 5 Department of Epidemiology, Colorado School of Public Health, University of Colorado, Anschutz Medical Campus, Aurora, CO 80045, USA; 6 Division of Pulmonary Sciences and Critical Care Medicine, School of Medicine, University of Colorado, Anschutz Medical Campus, 13001 E. 17th Pl., 3rd Floor, Mail Stop B119, Aurora, CO 80045, USA

**Keywords:** Total Worker Health, dissemination and implementation, RE-AIM, small business, consulting, safety and health

## Abstract

The role of dissemination and implementation (D&I) science is critical to the translation of *Total Worker Health*^®^ into practice and to the success of interventions in addressing current and future implications for worker safety, health, and well-being. D&I frameworks can guide researchers to design *Total Worker Health* (“TWH”) delivery approaches that use flexible implementation strategies to implement the core components of programs for employers with varying contextual factors, including small/mid/large-sized businesses and different industry types. To date, there have been very few examples of applying implementation frameworks for the translation and delivery of interventions into organizational settings that require adoption and implementation at the business level to benefit the working individuals. We present a TWH case study, Health Links™, to illustrate an approach to applying an existing implementation framework, RE-AIM, to plan, design, build, and then evaluate TWH implementation strategies. Our case study also highlights key concepts for scaling-out TWH evidence-based interventions where they are implemented in new workplace settings, new delivery systems, or both. Our example provides strong support of key implementation planning constructs including early and consistent stakeholder engagement, tailored messaging and marketing, flexibility, and adaptations in implementation strategies to maximize adoption, implementation, and maintenance among participating businesses.

## 1. Introduction

As chronic health conditions and disease rates continue to rise, attention has turned to the role employers play in supporting evidence-based interventions to improve worker health, safety, and well-being. *Total Worker Health^®^* (“TWH”) is a transdisciplinary approach for integrating workplace health promotion with occupational health and safety protection strategies promulgated by the National Institute for Safety and Health (NIOSH 2020). The approach represents key work and non-work domains of worker well-being including workplace policies and culture, work experience, safety climate, and health status [1]. Over the past decade, NIOSH and TWH researchers have developed frameworks and tools, such as the research-to-practice (r2p) approach, to help employers translate TWH into practice across a range of organizational settings [2]. r2p has focused on translating interventions to workplaces; however, it does not necessarily focus on the systematic study of these processes, as is customarily undertaken by D&I researchers [3]. The state of science of moving TWH research into practice has begun to shift to the application of dissemination and implementation (D&I) methods to improve translation [3,4,5,6]. The advances and rapid changes across the workplace, work, and workforce require special attention to the identification of the barriers and facilitators to adoption and implementation of TWH interventions [3].

This paper will present a case study, Health Links™, a TWH intervention aimed at improving the adoption and implementation of TWH in organizations across a range of industries, specifically targeting small- and mid-sized enterprises. In our approach for Health Links, we selected the RE-AIM (Reach, Effectiveness, Adoption, Implementation, and Maintenance) framework for designing and evaluating the program implementation because its flexibility and adaptability have been demonstrated across a range of settings [7].

### 1.1. TWH Intervention Background

TWH has its origin in the principles of organizational behavior change theory and the socio-ecological model used to identify key factors that influence worker health [8,9]. Conceptual models developed by the Centers of Excellence for *Total Worker Health^®^*, the Centers for Disease Control and Prevention (CDC), and the World Health Organization (WHO) present different approaches for program planning and implementation for workplace interventions. However, all underscore similar components of organizational support, leadership commitment, and employee involvement to inform the intervention components delivered and the ways that these components are delivered [10,11]. Businesses are increasingly seeking solutions for employee health, safety, and well-being that are evidence-based and can be operationalized in ways that are both practical and scalable [12,13].

The piece of “how” an intervention is delivered is defined as an “implementation strategy” in the field of D&I science [14]. Implementation strategies should be tailored to the local sociopolitical context [14]. In the case of TWH, it is common to identify organizations as a target setting for intervening to improve worker health, safety, and well-being and consider the unique needs these groups face [3,15]. Accordingly, implementation strategies are the methods by which certain core components are deployed to fit the varying priorities and needs of a business and its employees. The major categories of implementation strategies include stakeholder engagement, technical support for programs, the identification of business “champions”, and financial support for programs, among other strategies [16,17].

In support of this notion that we need to take a more comprehensive approach to identify the feasibility, quality, and scalability of TWH interventions, Bradley et al., published a summary from the December 2015 National Institutes of Health (NIH) Pathways to Prevention workshop “Total Worker Health-What’s Work Got to Do With It?” Bradley and coauthors recommended further D&I research to identify key factors that facilitate the “scaling out” of TWH and other evidence-based interventions across large networks of businesses/service systems [5]. In particular, scaling out includes implementing interventions in wide networks of businesses as well as organizations of different sizes and in different industry types. To address these calls for empirical data to inform how we scale out TWH with attention to the organizational context of businesses, D&I theories, models, and frameworks are foundational [18].

### 1.2. Application of D&I to TWH

D&I research has evolved over the past three decades to overcome pervasive challenges in translating evidence-based research into practice, including slow translation of evidence into practice, inequitable translation with low-resource settings being less likely to adopt evidence-based programs, and limited attention to key contextual factors that will facilitate or impede translation [7,19]. The D&I research literature includes theories, models, and frameworks that were empirically developed to accelerate the translation of evidence-based programs into practice [20]. Many of the theories and frameworks, including Roger’s Diffusion of Innovation and community-based participatory research (CBPR), have methodologically-rich literature indicating that shared-power and partnership with organizations and workers impact the ability of public health interventions to improve health behaviors [21,22]. As they relate to TWH, D&I frameworks provide opportunities to understand the modifiable organizational factors linked to the successful and sustainable translation of TWH. By targeting those factors with better implementation approaches and strategies for delivering TWH, D&I research holds promise to help more employers adopt and sustain TWH, thus leading to a greater impact on the health, safety, and well-being of working populations. Implementation frameworks can be broad and flexible in nature, spanning levels of the socio-ecological model [23]. The selection process involves considering key questions about the study, scope, timeframe, and availability of measures, and what level of the socio-ecological model is to be targeted [23].

The distinctions between D&I theories, models, and frameworks have been discussed in detail elsewhere and are beyond the scope of this paper [18,24,25,26]. In brief, they may be considered as process models that guide the steps of working with stakeholders to explore, plan, and implement an intervention; evaluation frameworks to assess implementation outcomes; and determinant theories/frameworks of constructs that predict implementation outcomes. Specifically, D&I frameworks can guide researchers to design TWH delivery approaches that use flexible implementation strategies to implement the core components of programs for employers with varying contextual factors, including small/mid/large-sized businesses and different industry types. While our case study will illustrate the application of these principles using the RE-AIM framework, we will return to the consideration of other models in the Discussion.

### 1.3. RE-AIM and Occupational Safety and Health

More than a hundred D&I theories, models, and frameworks exist and there are resources to search these to identify the appropriate guides for different implementation phases, such as planning, evaluation, or sustainment [27]. A smaller number of models or frameworks are useful across both the implementation planning and evaluation phases [3,7,24,28]. RE-AIM has been used for implementation planning and evaluation efforts for more than 20 years in a variety of settings. RE-AIM has evolved to include an iterative process for use in intervention planning, during implementation, as well as for program evaluation [29,30,31,32,33]. The Practical Robust Implementation and Sustainability Model (PRISM) further expands upon RE-AIM to allow for the conceptualization and specification of contextual factors [34,35].

The application of implementation frameworks to provide guidance on selecting implementation strategies to fit organizational contexts has been widely embraced in the design and delivery of interventions by clinical and community organizations [24,36] However, to date, despite their promise [3], there have been few examples of applying implementation frameworks for the translation and delivery of interventions into organizational settings that require adoption and implementation at the business level to benefit working individuals [4,15,37,38,39,40]. Specifically in relation to occupational safety and health translational research, it was noted that consideration of the unique and contextual challenges to achieving success in a workplace setting is critical in addition to finding a common language of D&I terminology [15].

As TWH researchers and practitioners design and implement workplace programs, we propose starting with two key questions regarding the influences of an employer’s and their teams’ decisions to deliver TWH in their workplace, or not. The first question is related to the characteristics of employers and their perspectives of TWH: “How do you apply implementation frameworks to successfully engage employers in TWH?” The second question is related to the organizational capacity for workplaces to both implement and sustain TWH: “How do you design TWH interventions to scale out successfully?” This paper offers a TWH case study to illustrate an approach to applying an existing implementation framework, RE-AIM, to plan, design, build, and then evaluate TWH implementation strategies with attention to these two questions.

### 1.4. Purpose

Our goal in this manuscript is to elevate the importance of using D&I frameworks to guide the implementation of TWH, through a case example of how we have used recent recommendations for the use of RE-AIM [7] to inform the planning/design and the evaluation of TWH interventions. Because RE-AIM has been more typically used in clinical and community settings, we highlight certain RE-AIM domains that warrant minor translation for the business organization context. Our case study describes a TWH intervention implemented at the organization-level and also highlights key concepts for “scaling-out” TWH evidence-based interventions where they are implemented in new workplace settings, new delivery systems, or both [41].

## 2. Application of RE-AIM to TWH Intervention Science

Applying RE-AIM to TWH Interventions

We selected the RE-AIM model because of its pragmatic use in planning and evaluation across multiple levels including community and organizations. It is broad yet flexible, allowing researchers to apply different dimensions of the framework based on the needs of the study [32]. While RE-AIM has been largely used in clinical settings to test and scale interventions directed at improving patient care, it has also been used in workplace-based interventions. For example, researchers evaluated a walking at work program in terms of the increasing of employee physical activity. In this study, they were able to use RE-AIM to measure the number of workplaces that participated in the program and, by evaluating its implementation, they found a range of activities delivered [42]. The adaptability of the framework lends itself to tailoring the evaluation process based on available resources/funding, and the scope of the project. RE-AIM principles may be applied across the discovery, planning, implementation, and evaluation stages of a project. This type of ongoing process review helps identify points where the delivery of a TWH intervention could be corrected, even in the slightest of ways, to promote the efficient implementation of the program, and to promote its long-term maintenance. Critiques of the use of the RE-AIM framework include limited information about how levels of the model interact; limited consensus on what constitutes adequate reach, adoption, and implementation; and the challenges involved in associating arbitrary time intervals [31]. These can be especially apparent when working in complex systems such as organizations [31].

The five dimensions of RE-AIM include Reach (the number and representativeness of participants), Effectiveness (the impact of the intervention on outcomes of interest), Adoption (the number and representativeness of settings and agents willing to implement a program), Implementation (the fidelity to the key components of the intervention), and Maintenance (the degree to which the program becomes institutionalized). To adapt to the unique needs of workplace adopters, we focused the five dimensions of RE-AIM so that they can be applied to the organizational-level settings of businesses. Table 1 specifically outlines the steps for TWH implementation mapped to the RE-AIM domains.

In the case of planning and implementing TWH interventions, adoption is defined in two ways: (1) the percent of businesses that agree to participate (setting level), and (2) the percent of staff that agree to participate (staff level). For workplace/organizational interventions, the setting and staff levels can be matched because the delivery agent representing the business is required to be the deciding factor in adoption. For example, we would measure adoption at the setting level by the number of businesses that adopt the TWH intervention equal to the staff level, which would be represented by an individual or team of people that are responsible for executing the changes in each organization. In another case, the setting-level and staff-level proportions could be different. For example, if there is an intervention targeting the adoption of a TWH training for managers to support employee mental health, the setting level would be the number of businesses that are willing to offer the training. The staff level would be the number of managers that participate in the training. There is an important distinction because TWH interventions often target multiple levels within an organization. Reach refers to the absolute number of employees that participate in TWH efforts (policies, programs, and supports) within a business. We discuss the considerations of how reach is defined and measured using RE-AIM below.

For RE-AIM, three important objectives of designing the implementation strategies/approaches and evaluating programs are: (1) to identify the key characteristics of adopters/end-users at both the setting and staff levels (target audience); (2) to determine the intervention perspectives of those adopters/end-users (i.e., priorities, facilitators/needs/resources, and barriers), (3) to understand the external incentives/penalties related to reach, adoption and implementation, and (4) to determine the likelihood of assessing the implementation and sustainability infrastructure—factors that will contribute to TWH implementation continuing after the external program facilitation/support has ended. For example, in this case study described below, our TWH intervention, Health Links™, provided external support for businesses to initially implement TWH, after which time employers could either sustain or terminate TWH delivery. The process for collecting multiple data points can involve qualitative and quantitative methods.

We also identified an opportunity to map RE-AIM constructs to the essential parts of the creation of a business plan. It turns out that there are many similarities that are relevant to how TWH interventions should gather information and design strategies that target employers as the primary “consumer” of TWH. The primary purpose of a business plan is to determine the concept and identify marketing and operational strategies for selling the product. This process involves understanding the needs and motivations of users, or in the case of RE-AIM, understanding how to reach and engage adopters. One could argue that in the TWH context, D&I is a public health product or service, and that business principles of product development, marketing, distribution, and finance can be translated for intervention design and implementation strategy (Brownson 2017). These business plan sections are also outlined in Table 1 and described in the case study below.

## 3. The Health Links^®^ Case Study

The Health Links program was developed and launched in 2013. It was designed applying RE-AIM in concert with theories of health behavior and organizational change as an intervention to maximize participation in TWH among employers as a means for improving worker health, safety, and well-being. The program has three main intervention components: (1) an assessment tool that helps an organization self-evaluate across six benchmarks (Organizational Supports, Workplace Assessment, Health Policies and Programs, Safety Policies and Programs, Engagement, and Evaluation), (2) one-on-one advising sessions that assist organizations in goal setting for TWH improvements in areas of greatest need, and (3) access to TWH evidence-based resources including implementation aides developed by NIOSH and other Centers of Excellence for TWH. When the program launched, our first goal was to first establish its effectiveness before scaling it to reach many employers, specifically small businesses. To achieve that, we tested its effectiveness through a pilot study in Pueblo, Colorado [37]. After establishing effectiveness, we conducted a process of stakeholder engagement to identify implementation strategies for delivering an intervention that would appeal and function across diverse workplace settings, was adaptable to meet unique business needs, and that could be implemented by a variety of people in an organization.

### 3.1. Implementation Design

The multi-level TWH conceptual approach for the Health Links program applies organizational behavior change and dissemination theories. According to the socio-ecological model, individual behavior change is based on influences across self, relationship, community (in this case, including organization), and society [9]. We aimed to reach individuals responsible for TWH within organizations in order to influence workplace conditions that in-turn influence organizational actions that would protect and promote worker health and safety.

**Figure 1:**
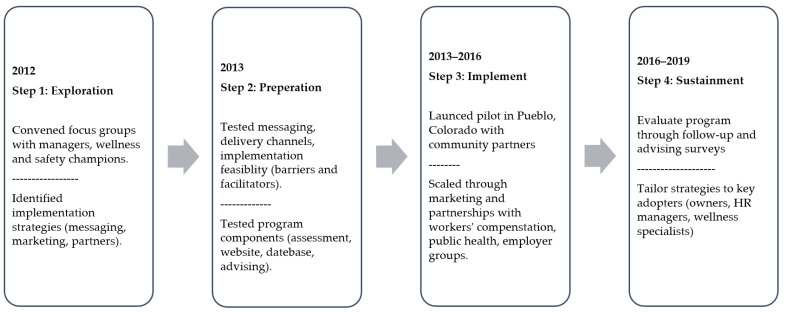
Timeline of Health Links implementation steps.

The Health Links intervention followed a D&I process model approach that can be illustrated in four phases, as shown in Figure 1: exploration, preparation, implementation, and sustainment. We initially identified our program adopters, explored their priorities/needs related to the contextual factors that predict RE-AIM outcomes, planned the implementation armed with this stakeholder input, and then implemented the program [18,43]. We convened a multidisciplinary team of communication specialists, public health practice professionals, and occupational safety and health experts to guide our implementation process. First, we wrote a business plan for Health Links, to assess the landscape of workplace health promotion and health protection programs, identify the stakeholders who would serve as program adopters and implementers, state outcomes and performance objectives, select and design implementation strategies, and develop a communication and marketing plan, including a dissemination arm that was charged with identifying channel partners to conduct outreach in communities of business. We next conducted focus groups and key informant interviews with business leaders, small business owners, employees, chambers of commerce, insurers, brokers, offices of economic development, marketing and advertising consultants, public health officials, experts in occupational health and safety, occupational medicine, health promotion practitioners, and other stakeholders. The goal was to consider multi-stakeholder perspectives to ensure the most relevant intervention components were incorporated into our program. The major themes that were identified from the focus groups and interviews determined that the intervention must: (1) be based on best available evidence, (2) accommodate the needs of many different types of businesses and workforces, (3) be feasible for small businesses to access and adopt, meaning inexpensive and not resource/time intensive, (4) be scalable to large numbers of businesses, (5) apply basic principles of organizational change management, and (6) generate metrics so that the program could be evaluated in five domains of the RE-AIM model: reach, efficacy, adoption, implementation, and maintenance of the intervention [32].

Through stakeholder input, we established the Health Links program’s core intervention package, including: (1) the online Healthy Workplace Assessment to benchmark existing TWH policies and practices and identify strengths and opportunities for improvement in an organization, (2) one-on-one advising to provide assistance and coaching for TWH goal setting by the organization, and (3) certification to recognize achievement in TWH in three levels (Certified Healthy Workplace, Partner, and Leader). The Health Links Healthy Workplace Assessment™ was developed by adopting measures from the CDC Healthy Workplace Scorecard, the WHO Healthy Workplace Framework, and the TWH framework to measure policies, programs, and workplace strategies across six benchmarks: Organizational Supports, Workplace Assessment, Health Policies and Programs, Safety, Engagement, and Evaluation.

Advising was determined to be particularly important in the provision of ongoing consultation to organizations and their TWH delivery agents to identify goals and evidence-based strategies and resources to use [38]. During the advising process, representatives from organizations met with Health Links advisors to review the results of the organization’s Healthy Workplace Assessment and participate in on-site and virtual (live telephonic or video) sessions. During these sessions, the advisor worked with the designated representatives from the organization to identify strengths and barriers to improving TWH and created action plans that set three SMART (specific, measurable, actionable, relevant, and timely) goals, each with action steps for putting evidence-based strategies in place. Principles from theories of motivational interviewing were incorporated to serve as a technique for addressing the unique needs of the organization and their workers.

### 3.2. Implementation Strategies

We used RE-AIM principles with stakeholders during the exploration and planning stages to formulate implementation strategies that would target the key priorities, address facilitators and barriers that relate to the characteristics and perspectives of end-user organizations and workers, and develop an implementation and sustainability infrastructure. The process maps to the expert recommendations for implementing change (ERIC) steps for planning implementation strategies, which also demonstrates how using RE-AIM in conjuncture with other D&I frameworks is beneficial [44]. The steps below describe the key ERIC concepts for pre-implementation, active implementation, and sustainment built on (1) gathering information to identify needs and barriers, (2) selecting and tailoring strategies that are modeled to scale up, and (3) building buy-in from partners and stakeholders.

### 3.3. Message Development

Our primary audience for communications about the Health Links intervention were delivery agents of TWH. These individuals were identified as representatives responsible for employee health, safety, and well-being in organizations. The communication challenge is that this audience is diverse, including business owners, managers, human resource professionals, health and safety professionals, and volunteer employee committees designated to run wellness committees. We developed messaging and program content aimed at encouraging employers (by way of individuals or groups) to take the necessary action to sign up and participate in Health Links. Feedback gathered from the focus groups and pilot informed the content presented on the website [45] and optimized the user experience. Another communication challenge was that we needed to educate our audience about TWH as well as provide information on how to participate. We developed messaging that provided basic information on the benefits of TWH, including improving employee health, safety, and productivity; reducing absenteeism and presenteeism; and being an employer of choice. The final messaging concisely communicated how to join the program. The final website included basic information on the TWH approach, easy steps to begin, examples of the assessment, success stories that showcased Health Links businesses, and a simple call to action to “Get Started”. The website also includes a resource center, a training and events page, and a dashboard where businesses can view their organizational assessment, report card, and schedule advice, and can download action plans and access resources and modules that provide deeper dives into each benchmark and links to specific topic areas including family-friendly workplaces, stress management, leadership support, and employee engagement [45]. Figure 1 illustrates the core implementation strategies including direct outreach to businesses and indirect dissemination through partners.

### 3.4. Dissemination

To establish our approach to dissemination, we applied concepts from RE-AIM and business planning as outlined in Table 1. Dissemination strategies are a subset of implementation strategies that particularly attend to the natural communication channels of end-users and developing networks of individuals—TWH champions who share our priorities for implementation [20]. Commonly, these TWH champions are representatives who already have health and safety responsibilities within organizations. They were quick to understand the TWH concept and to value the importance of promoting employee health. For implementation, we define these individuals as the delivery agents. They are the ones that are responsible for adopting and implementing Health Links as a program that benefits the organization and, by way of workplace policies and programs, individual employees. In addition, partners including chambers of commerce, small business and economic development centers, public health agencies, and insurance groups in healthcare and workers’ compensation, were identified as our secondary target audience. These groups were previously identified as “intermediaries” and were specifically found to be effective when working to engage small businesses [15,46]. These groups served as our dissemination partners and were considered key in program adoption. Engaging dissemination partners involved relationship building with program directors, community outreach and relations staff, and health educators that had an agenda for delivering public health programming to employers. Chambers of commerce were identified by stakeholders as a trusted source of information, serving as a business network, a local organization, whose goal is to further the interests of businesses. They also represent members from different trades and industries, which was a helpful way of broadening representation across businesses. Local public health agencies were eager to partner with Health Links as a vehicle to deliver workplace-based programs for health promotion addressing tobacco cessation, disease prevention and management, and breastfeeding accommodations. We worked with partners to develop co-branded communication materials including newsletters, event promotions, and training flyers. Ways in which we adapted these delivery methods to increase adoption and scale the program are described below. By disseminating through business groups and trade associations, we aimed to engage healthcare, education, municipal, construction, and service industries.

To communicate the success of the program to stakeholders, we targeted the contextual influence of an employer’s perspective on the intervention [7,34,47]. We invited participating businesses to share success stories through interviews and online blogs that highlighted how they were implementing TWH and how their participation in Health Links as an intervention had influenced positive change in their organization. We collected video and written testimonials to share personal stories and successes about Health Links to inspire businesses to act and join Health Links. We found that communication among the participants was also important to provide evidence of the intervention’s effectiveness to employees and decision-makers. Developing the communication tools for businesses to share their TWH successes was, therefore, identified as an important implementation strategy that we promoted in recruitment, during participation, and during retention efforts. These tools included a short press release, sample social media posts, and certification badges that could be shared through the business’s communications and on their websites.

### 3.5. Adaptation

Over the course of seven years, we evaluated and then adapted the implementation blueprint for Health with the goal of enhancing employer adoption and honing our implementation strategies to optimize its scale out. This approach of conducting process and intermediary outcome evaluations is analogous to what others have undertaken when applying RE-AIM during program implementation [29]. Two significant changes included updating the Healthy Workplace Assessment and transitioning advising from in-person community advisors to a virtual model. During the initial four years, we offered two versions of the Assessment based on levels of organizational readiness. One version was for organizations that met the criteria for Healthy Workplace Certification. The other version was for those that were aspiring to reach, but not yet meeting, qualifying criteria. After learning that the two pathways were creating confusion for users, we created a single version of the assessment with updated questions and an improved user journey. In revising the survey tool, we designed a stepped process for each section and explanatory text for each question. We verified responses through advising to track and evaluate how businesses were implementing new TWH policies and programs annually [38].

**Figure 2:**
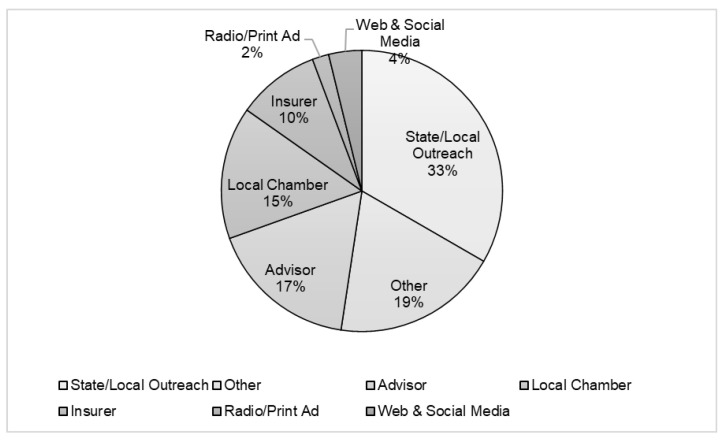
Primary sources of dissemination indicated by Health Links enrolled users. “How did you hear about Health Links?”.

In 2016, we conducted a pilot in Oregon in partnership with a state-based workers’ compensation insurer to test Health Links in a new market. We implemented advising through Zoom to test a virtual model as a next step for scaling the program nationally and to offer greater reach to employers where there were no local Health Links community advisors. The pilot resulted in the decision to offer virtual advising exclusively as part of the program’s expansion. Community advisors brought face-to-face connection to businesses and a local, personal touch, and were able to apply direct outreach methods for dissemination and recruitment, as indicated in Figure 2. However, the tradeoff was that managing a team of 12 remote advisors required a full-time staff person, ongoing training, financial and contract support to compensate them, and a continuous effort to retain current and recruit new advisors. The actual program cost per participating business was compared to the expense of hiring a full-time in-house advisor with the responsibility of providing double the number of advising sessions.

We also adapted our communication strategies based on input from our stakeholders and dissemination partners. We modified messaging and marketing to be able to co-brand program materials as well as speak directly to specific audiences. For example, when partnering with trade associations such as the Colorado Motor Carriers Association, we co-wrote a letter to members with their president that included basic information on the program and TWH and how it would benefit truck drivers. We also learned to leverage the reach of existing public health efforts in the region. When partnering with the state health department to engage small employers, we incorporated information about the benefits of businesses promoting the National Diabetes Prevention Program in Health Links outreach. Importantly, when we were delivering Health Links in different regions of the state, we aligned with the community health goals and held Health Links trainings based on the local needs of employers. These examples illustrate a successful way to raise awareness about TWH and promote Health Links as a valuable program while establishing relationships with local partners.

After making these mid-course adjustments, we observed better uptake demonstrated by expanded adoption among employers. We were able to scale the delivery of the TWH intervention because our implementation strategies appealed to a broader range of organizations. The types of adaptations that we needed to make to implement the program were both proactive (planned-in-advance) and reactive (agile adjustments to address unanticipated barriers). These types of adaptations can be categorized under RE-AIM as changes to who delivered the program (target audiences and distribution strategy) and implementation strategies to scale the program including tailored messaging, improved functionality of the web portal to deliver core intervention components, and promoting the program more broadly.

### 3.6. Scaling Out

We saw an opportunity to expand Health Link’s adoption by employers after three years. Participants of the first generation of Health Links (2013–2016) provided feedback in the form of 6-month follow up surveys, giving evaluations of advising sessions and partner meetings. They identified advising sessions and organization recognition as the two most valuable components of the program. Respondents of the advising survey (*N* = 170) rated advising on a 5-point scale as helpful (4.62) and reported that they were confident to start or improve their wellness and safety program (4.52). Participants representing Health Links-certified businesses shared in a focus group (*N* = 12) that they viewed the Health Links brand as having a favorable reputation, found it to be a credible evidence-based program, and did not find cost to be a barrier to participation. However, some participants felt the website was hard to navigate, reporting that it took too many steps online to begin participating. Therefore, we re-tooled parts of the intervention and addressed some implementation barriers in the next generation of Health Links in 2016, using these program evaluation data to (1) enhance the online user experience, (2) create a plan structure for participation that allowed businesses to select the level of access and number of advising sessions, and (3) increase the reference and overall terminology to TWH in our messaging.

In the next generation of Health Links, we improved the user experience for a more focused primary audience: decision-makers in organizations including executives, managers, human resource professionals, and health and safety professionals. The messaging spoke to these still very diverse audiences by identifying common roles, values, and motivations. It presented a vision of TWH as the umbrella for delivering occupational health and safety and sharing the alignment with our program’s mission. We found it important to walk a fine line by showing that Health Links is practical as well as evidence based. We used language that reflected our academic underpinnings, while staying approachable and adaptable to organizations that ranged in industry and size. The visual components of the delivering Health Links were also important to portraying the look and feel of TWH. We selected images of diverse workers—individuals in their work environments that differed in age, race, ethnicity, and geographical location. The branding for Health Links overall was fresh, fun, and modern.

## 4. Evaluation Methods

We developed methods to evaluate Health Links’ three domains of RE-AIM: adoption, implementation, and maintenance.

To estimate adoption, we measured the number of businesses that enrolled in Health Links. We were able to estimate the proportion of those that committed versus those that were in the precontemplation phase of adoption by measuring the number of businesses that expressed interest by way of a consult and/or registration. In the planning phases of the intervention, we identified primary barriers to participation through formative research and continued this evaluation process by direct business outreach conducted through phone calls and emails with primary contacts. We deployed adaptation strategies throughout the intervention to address the barriers to enhance participation as described below. We collected and tracked data from implementation strategies for marketing the program including trainings, email and print marketing, social media, and direct outreach through community advisors and program staff. To understand the how and why of adoption, we included a question on the user registration page to ask “how did you hear about us?” in order to understand what methods were most successful in reaching user groups. The counts for reach were based on multiple touch points that represented a single individual or multiple delivery agents from the same organization receiving the information. For our denominator, we used county-level data to estimate the number of businesses in our target market for those counties where we were disseminating program information and had positioned channel partners. There were no exclusion criteria for participating in Health Links to consider. We also recorded reasons why participating businesses decided not to participate in a subset of pilot businesses.

**Figure 3:**
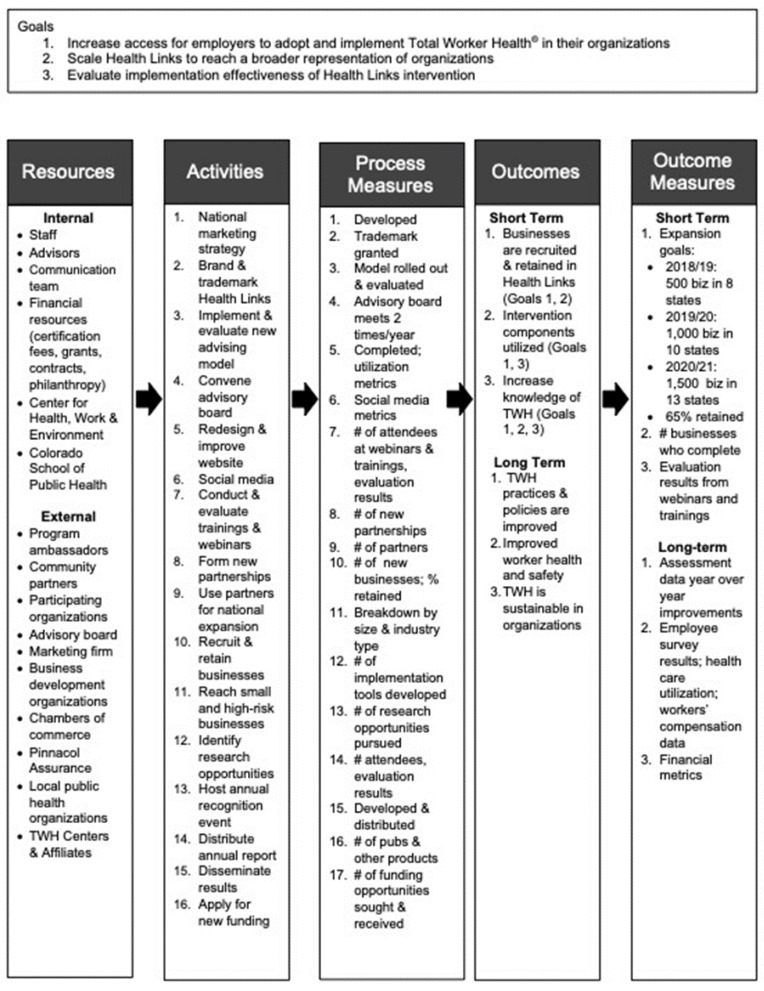
Logic model for Health Links’ intervention implementation design.

To measure implementation, we first developed a logic model to identify and describe the relationships between inputs, activities, outputs, and implementation outcomes (Figure 3). We administered a 6-month follow up survey to obtain feedback on how businesses were implementing TWH and to gauge the extent to which primary contacts from the businesses were following through on the goals they set in the Health Links advising sessions. Our program staff also tracked the businesses that had higher versus lower levels of program use by assessment completion rates, advising rates, and program attrition. We did not collect cost information from organizations and, therefore, were not able to calculate the implementation cost for each business. This is an important area for future work. We did attempt to deliver a low time burden intervention, based on our initial stakeholder input.

To estimate maintenance, we continued to contact participating organizations after dropout. Through surveys and business relations, we debriefed with business representatives to identify what they liked best and least about the program and which aspects they were interested in continuing and modifying. For dropout businesses, we employed follow-up emails and phone calls to qualitatively understand reasons for not continuing to utilize the intervention. We also used indicators of program-level maintenance including retention rates, re-enrollment lapses, and changes to participation levels. Because Health Links is an ongoing service, as opposed to a research study with an end date, a number of these evaluation practices are embedded in the program as quality assessment and quality improvement practices. These measures map directly to the Maintenance planning tools promoted by the RE-AIM framework (RE-AIM Measures and Checklist 2019).

## 5. Discussion

Our experience in applying RE-AIM as the implementation framework for Health Links led to a few major conclusions. First, the approach to guiding the process of translating TWH evidence into practice and evaluating implementation outcomes was iterative and continuous. If research on a TWH intervention shows efficacy, investigators must be prepared to make a major transition in translating a promising TWH product or service into an ongoing, sustainable intervention. The Health Links case study illustrates the relevance of selecting an implementation framework for planning, delivering, and evaluating TWH interventions and the merits of how implementation frameworks can inform the program logics for converting promising research into ongoing practice.

Establishing relationships with partners and stakeholders to identify the complex set of factors that impact TWH adoption and implementation proved crucial. By measuring adoption and maintenance, we determined that employers who are interested and motivated to participate will continue to participate in a TWH intervention, especially if it is practical, evidence based, and returns value for the time and cost invested by the organization. Priming action of these organizations requires the identification of both the individuals responsible for employee health and safety and the decision makers who consider TWH a medium-to-top priority. Many organizational staff are not familiar with TWH, so it proved to be necessary to educate target organizations and present the case for why TWH holds promise for improving their approach to health and safety. In effect, we learned that the application of principles from community participatory-based research should be integrated into TWH intervention frameworks.

The EPIS (Exploration, Preparation, Implementation, Sustainment) framework that we used in our timeline (Figure 1) highlights the importance of engaging stakeholders from multiple levels to inform and influence system-driven implementation efforts [48]. This notion parallels business and product development models that stress the importance of market research to engage users and consumers in the process of informing what is desirable and functional. Identifying barriers and facilitators was critical to planning how to reach employers and increase program adoption. Gathering feedback from decision makers in organizations to understand motivations for TWH also aids in planning and delivering effective interventions. Operationalizing TWH into any business setting may require the breaking down of silos due to the nature of how employee health and safety is implemented. It involves coordination across management, HR, communications, marketing, and health and safety.

Crafting an implementation strategy that is ‘tailored to context’ is extremely important. Businesses, similarly to people, have periods when they are more receptive to new initiatives and new behaviors. Businesses operate on many different calendars, with different production/service peak times, times when they decide upon new workplace health and safety initiatives and allocate resources (time and budget). As such, there are times when organizations will be more receptive to a TWH intervention. In some cases, the acknowledgement that TWH may not be a top priority informs the delivery of the program itself. For example, some organizations we worked with had signing up for Health Links on their list but waited to right before the holidays to enroll while others decided to act during slower business periods. Tracking enrollment and reenrollment trends helped us pinpoint adjustments for our outreach and dissemination activities. Re-prompting organizations to implement Health Links, especially during the organization’s ‘down time’ periods, is a useful strategy for recruitment.

Consistent with D&I research, it is important to craft the intervention to fit into a ‘blanket’ implementation strategy of tailoring to the context of the adopting organization [19]. In making the transition from research to practice, investigators must be prepared to adapt the intervention based on ongoing evaluation and be agile in deriving creative solutions. Our example supports previous proposals for D&I science to consider adaptations that go beyond cultural elements [49,50]. Health Links required modification to implementation strategies to fit different employer characteristics, organizational contexts, and even community- and industry-based settings. Akin to continuous quality improvement practices, having a continuous process of asking key questions to identify what organizations and decision makers valued about TWH and Health Links has proven to be formative. Understanding the level of TWH knowledge and expertise in an organization and building relationships with participating businesses and intermediaries all served as important ways to improve program adoption and implementation. We gained real-time feedback from stakeholders to test our implementation approaches by surveying our established network of businesses.

Frequent and disciplined evaluation practices provide important lessons about the role of communication. Marketing TWH to employers required a mix of state and local outreach, staff outreach, and training and supporting local advisors to conduct recruitment. For Health Links, the three most influential partners in implementation were chambers of commerce, local public health agencies, and workers’ compensation insurers. These groups should be considered as effective channels for sharing TWH information and implementing programs, depending on the target of the intervention. We also learned that certain content appealed to different audiences within organizations. Some businesses and individuals were more interested in Healthy Workplace Certification and recognition, while others reported more value in the advising sessions. The range of motivations required a strategy for implementation that can appeal to a wide range of individuals representing different roles including ownership, management, human resources, and health and safety.

Our experience illustrates why TWH interventions require the efforts of a multidisciplinary team that is prepared to integrate its knowledge to create a product or service that can integrate with business systems. In the Health Links example, forming a program team with expertise in program planning, health communication, marketing, sales, statistics, psychology, health promotion, occupational health and safety, and business development allowed for diverse viewpoints on program components and dissemination methods. This is also consistent with recommendations for using implementation frameworks that stress continuous cycles of establishing and maintaining stakeholder engagement, exploration to determine implementation factors, selecting and tailoring implementation strategy(s), and then repeating the cycle with attention to sustainability considerations [43,51].

For the Health Links intervention, RE-AIM proved to be an adequate implementation framework for designing and evaluating an intervention in accordance with the RE-AIM domains of Adoption, Implementation, and Maintenance, and for conducting repeated domain-specific evaluations to guide continuous process improvement. We experienced several limitations to identifying and measuring RE-AIM dimensions. Specifically for Reach, we do not have outcome measures on individual workers who benefited from businesses adopting and implementing Health Links. However, from the perspective of occupational safety and health purveyors of TWH, we submit for consideration that one might also define the number or percentage of businesses who benefitted from our outreach sufficiently recruit businesses as our Reach to workplaces—even though it is more conventional in RE-AIM to define this term as Adoption. This point raises a debate for D&I and occupational safety and health around how to define and measure reach in the application of RE-AIM to organizational-level interventions that have the primary purpose of changing workplace behaviors. Measuring the percentage of businesses that agree to adopt TWH can also be challenge for wide-reaching TWH interventions because while we may know the numerator of adopting organizations, it is not always feasible to estimate a denominator of businesses that had heard about, and thus had the opportunity to adopt, the intervention, when using multiple marketing channels to disseminate an intervention at the organizational level. Furthermore, because businesses learned about Health Links in different ways, we may have or may not have been able to connect that encounter to participation.

Additionally, there are contextual factors that influence the way Health Links is implemented by organizations. As described in the case study, these factors included the different types of adopters, who vary in their motivations, roles, and influence within the organization. These attributes, among others, likely impact the level of engagement. We know there are many other internal and external influences that cannot always all be measured, which may limit generalizability.

While this paper focused on RE-AIM, we acknowledge that there are many other D&I theories, models, and frameworks that could be used at a business setting for TWH interventions [3]. One of the benefits of using the RE-AIM framework is measuring the mediating relationships between implementation strategies and implementation outcomes and the ability to track and adapt over time. There have been models developed to look at this specifically. The Practical, Robust, Implementation, and Sustainability Model (PRISM) expands upon RE-AIM and contains constructs to understand the contextual factors that exist between the intervention characteristics and the ecosystem where implementation occurs [34]. We were able to track some of this, in part, by collecting information on barriers and facilitators to adoption and implementation through Health Links advising, focus groups, and interviews. PRISM also provides a way to enhance maintenance at the work setting level by building in pieces of the intervention to organizational infrastructure such as job requirements, audits, and institutionalization. This type of real-time evaluation and program improvement process is the future of research-to-practice. New D&I models have promise, especially in TWH intervention research that involves multiple and complex implementation targets across work settings.

Future research for TWH implementation science will need to address gaps in how TWH is adapted and operationalized in the real-world where businesses are constantly facing changing demands. This will require that translation activities start at the earliest stages of development and continually assess barriers and facilitators to adoption and maintenance over time [15]. The ideal scenario for evaluating the implementation of any TWH intervention would be to apply a systems approach to assess how organizations and individuals responsible for TWH deliver and tailor it, and why. Glasgow et al. argue that a program cannot be successful if it is effective solely at the individual level because it must be able to be adopted and implemented consistently in a variety of settings and by a variety of agents [7,30]. More research is needed to fully understand organizational factors and the measurement of organizational-level impact as a result of TWH in practice. Formative research including focus groups and stakeholder engagement can assist in identifying the primary and secondary audiences for TWH interventions. Marketing research methods can also help assess the demand for TWH among business communities and determine the existing competition. These are all important aspects of D&I for ensuring that TWH programs are differentiated for maximizing reach and adoption. There are also business models such as Rogers’ Diffusion of Innovation and Segmentation, Targeting and Positioning (STP) [22,52] that provide similar approaches for conceptualizing dissemination and implementation through the lends of marketing and product development. More coordination between business institutions and TWH may be beneficial to develop blended models for implementation science in business settings.

## 6. Conclusions

In conclusion, as TWH research expands to consider applied implementation science, the RE-AIM framework provides a strong model to inform the planning and evaluation of TWH interventions. Through Health Links, we demonstrated the importance of flexibility in the application of implementation frameworks to accommodate adaptation in design and objectives, in general, and for TWH interventions specifically.

## Figures and Tables

**Table 1:** Implementation outcome measures by RE-AIM component applied to organizational-level TWH interventions.

RE-AIM Outcome Dimension	Definition	Business Planning	Outcome Metrics for Organizational/Business Setting	Steps for Implementation	Evaluation Considerations
**Adoption level 1**—organizations contemplating participation after initial contact	The number of businesses and the number of delivery agents that are willing to consider participation	*Marketing* (Target audience, competitive analysis, positioning, pricing, messaging.)	The number of businesses that receive information about the TWH intervention through implementation strategies including communication, marketing, direct outreach, and stakeholder engagement.	Build relationships with stakeholders and partners across multiple levels of implementation ecosystem. Solicit stakeholder feedback on implementation strategies. Allow for new ideas, modifications, and revisions from users.	Defining the target number (denominator) and representativeness of organizations. Not pragmatic to go back historically and estimate.
**Adoption level 2**—organizations sign up to deliver TWH	The number of businesses and the number of intervention agents (people who deliver TWH) who are willing to initiate program.	*Distribution, sales strategy* (Moving the product from conception to end user.)	Setting level—The number of businesses that enroll or sign-up in the TWH intervention. Enrollment rate by groups (business size, industry, geographical location), company supports for TWH. Agent level—Percent of employees that participate. Characteristics of employees participating vs. nonparticipating employees.	Identify decision makers and the core values/mission of organization. Identifying existing practices, including expertise of delivery agents, to implement TWH programs. Provide data and information (success stories, testimonial) on effectiveness of program.	Can be defined as the number that are willing to initiate or implement programs compared to the number offered and why.
**Implementation** Fidelity Adaptations	The extent to which the intervention or program happened as intended in the business.	*Product testing* (Product review, product changes, costs.)	The number of organizations that complete the TWH intervention components. The adaptions to program deliver (who delivers it, changes to materials, customizations) The cost of staff time to complete assessment and advising to improve TWH.	Use a participatory process with stakeholders to identify barriers and facilitators to implementation. Tailor implementation strategies to level of experience of target audience. Develop and refine evaluations questions related to needs, process, effectiveness, and associated outcomes. Develop logic model to describe relationships between inputs, outputs, and implementation outcomes.	Characterize adaptations, type of adaptation, and why it was made at both the intervention level and the organizational level. Calculate the cost of the implementation effort at different levels (intervention and organization).
**Maintenance**	The extent to which TWH becomes institutionalized within the organization’s operation or becomes a routine practice and policy.	*Sustainability* (Loyal customer base, revenue neutral, and profitability.)	The number of businesses that still have implemented intervention (TWH) at ≥6 months post intervention. TWH is aligned and included in organization’s mission and/or business model.	Build TWH into existing infrastructure. Conduct check-ins with businesses that encourage TWH progress. Focus on environmental or policy changes.	Measure long-term effects of program by TWH outcome. Information that is available on long-term institutional efforts.

## References

[1] Chari R., Chang C.-C., Sauter S.L., Sayers E.L.P., Cerully J.L., Schulte P., Schill A.L., Uscher-Pines L. (2018). Expanding the Paradigm of Occupational Safety and Health. J. Occup. Environ. Med..

[2] NIOSH r2p. https://www.cdc.gov/niosh/r2p/default.html.

[3] Guerin R.J., Harden S.M., Rabin B.A., Rohlman D.S., Cunningham T.R., TePoel M.R., Parish M., Glasgow R.E. (2021). Dissemination and Implementation Science Approaches for Occupational Safety and Health Research: Implications for Advancing Total Worker Health. Int. J. Environ. Res. Public Health.

[4] Anger W.K., Elliot D.L., Bodner T., Olson R., Rohlman D.S., Truxillo D.M., Kuehl K.S., Hammer L.B., Montgomery D. (2015). Effectiveness of Total Worker Health interventions. J. Occup. Health Psychol..

[5] Bradley C.J., Grossman D.C., Hubbard R.A., Ortega A.N., Curry S.J. (2016). Integrated interventions for improving Total Worker Health: A panel report from the National Institutes of Health Pathways to Prevention Workshop: Total Worker Health—What’s Work Got to Do with It?. Ann. Intern. Med..

[6] Tamers S.L., Chosewood L.C., Childress A., Hudson H., Nigam J., Chang C.-C. (2019). *Total Worker Health*^®^ 2014–2018: The Novel Approach to Worker Safety, Health, and Well-Being Evolves. Int. J. Environ. Res. Public Health.

[7] Glasgow R.E., Harden S.M., Gaglio B., Rabin B., Smith M.L., Porter G.C., Ory M.G., Estabrooks P.A. (2019). RE-AIM Planning and Evaluation Framework: Adapting to New Science and Practice With a 20-Year Review. Front. Public Health.

[8] Burton J., World Health Organization (2010). WHO Healthy Workplace Framework and Model: Background and Supporting Literature and Practices.

[9] Centers for Disease Control and Prevention The Socio-Ecological Model: A Framework for Prevention. https://www.cdc.gov/violenceprevention/publichealthissue/social-ecologicalmodel.html.

[10] Harris J.R., Hannon P.A., Beresford S.A., Linnan L.A., McLellan D.L. (2014). Health promotion in smaller workplaces in the United States. Annu. Rev. Public Health.

[11] Schwatka N.V., Tenney L., Dally M.J., Scott J., Brown C.E., Weitzenkamp D., Shore E., Newman L.S. (2018). Small Business Total Worker Health: A Conceptual and Methodological Approach to Facilitating Organizational Change. Occup. Health Sci..

[12] Dugan A.G., Punnett L. (2017). Dissemination and Implementation Research for Occupational Safety and Health. Occup. Health Sci..

[13] Hudson H.L., Nigam J.A.S. (2019). Future directions and opportunities for Total Worker Health^®^. Total Worker Health.

[14] Frieden T.R. (2014). Six Components Necessary for Effective Public Health Program Implementation. Am. J. Public Health.

[15] Cunningham T., Jacklitsch B., Richards R. (2021). Intermediary Perspectives on Total Worker Health in Small Businesses. Int. J. Environ. Res. Public Health.

[16] Kirchner J.E., Waltz T.J., Powell B.J., Smith J.L., Proctor E.K. (2017). Implementation strategies. Dissemination and Implementation Research in Health: Translating Science to Practice.

[17] Mazzucca S., Tabak R.G., Pilar M., Ramsey A.T., Baumann A.A., Kryzer E., Lewis E.M., Padek M., Powell B.J., Brownson R.C. (2018). Variation in Research Designs Used to Test the Effectiveness of Dissemination and Implementation Strategies: A Review. Front. Public Health.

[18] Nilsen P., Bernhardsson S. (2019). Context matters in implementation science: A scoping review of determinant frameworks that describe contextual determinants for implementation outcomes. BMC Health Serv. Res..

[19] Balas E., Boren S. (2000). Managing Clinical Knowledge for Health Care Improvement. Yearb. Med. Inform..

[20] Brownson R.C., Colditz G.A., Proctor E.K. (2012). Dissemination and Implementation Research in Health Translating Science to Practice. Dissemination and Implementation Research in Health: Translating Science to Practice.

[21] Cook W.K. (2008). Integrating research and action: A systematic review of community-based participatory research to address health disparities in environmental and occupational health in the USA. J. Epidemiol. Community Health.

[22] Lundblad J.P. (2003). A review and critique of Rogers’ diffusion of innovation theory as it applies to organizations. Organ. Dev. J..

[23] Tabak R.G., Chambers D.A., Hook M., Brownson R.C. (2018). The conceptual basis for dissemination and implementation research. Dissemination and Implementation Research in Health: Translating Science to Practice.

[24] Damschroder L.J., Aron D.C., Keith R.E., Kirsh S.R., Alexander J.A., Lowery J.C. (2009). Fostering implementation of health services research findings into practice: A consolidated framework for advancing implementation science. Implement. Sci..

[25] Damschroder L.J. (2019). Clarity out of chaos: Use of theory in implementation research. Psychiatry Res..

[26] Nilsen P. (2015). Making sense of implementation theories, models and frameworks. Implement. Sci..

[27] Dissemination and Implementation Models in Health Research and Practice. www.dissemination-implementation.org.

[28] Nilsen P., Birken S. (2020). Handbook on Implementation Science.

[29] Glasgow R.E., Battaglia C., McCreight M., Ayele R.A., Rabin B.A. (2020). Making Implementation Science More Rapid: Use of the RE-AIM Framework for Mid-Course Adaptations Across Five Health Services Research Projects in the Veterans Health Administration. Front. Public Health.

[30] Glasgow R.E., Lichtenstein E., Marcus A.C. (2003). Why Don’t We See More Translation of Health Promotion Research to Practice? Rethinking the Efficacy-to-Effectiveness Transition. Am. J. Public Health.

[31] Glasgow R.E., Klesges L., Dzewaltowski D., Estabrooks P.A., Vogt T.M. (2006). Evaluating the impact of health promotion programs: Using the RE-AIM framework to form summary measures for decision making involving complex issues. Health Educ. Res..

[32] Glasgow R.E., Vogt T.M., Boles S.M. (1999). Evaluating the public health impact of health promotion interventions: The RE-AIM framework. Am. J. Public Health.

[33] Harden S.M., Smith M.L., Ory M.G., Smith-Ray R.L., Estabrooks P.A., Glasgow R.E. (2018). RE-AIM in Clinical, Community, and Corporate Settings: Perspectives, Strategies, and Recommendations to Enhance Public Health Impact. Front. Public Health.

[34] Feldstein A.C., Glasgow R.E. (2008). A practical, robust implementation and sustainability model (PRISM) for integrating research findings into practice. Joint. Comm. J. Qual. Patient Saf..

[35] McCreight M.S., Rabin B.A., Glasgow R.E., Ayele R.A., Leonard C.A., Gilmartin H.M., Frank J.W., Hess P.L., Burke R.E., Battaglia C.T. (2019). Using the Practical, Robust Implementation and Sustainability Model (PRISM) to qualitatively assess multilevel contextual factors to help plan, implement, evaluate, and disseminate health services programs. Transl. Behav. Med..

[36] Gaglio B., Shoup J.A., Glasgow R.E. (2013). The RE-AIM Framework: A Systematic Review of Use Over Time. Am. J. Public Health.

[37] Tenney L., Fan W., Dally M., Scott J., Haan M., Rivera K., Newman M., Newman L.S. (2019). Health Links™ Assessment of Total Worker Health^®^ Practices as Indicators of Organizational Behavior in Small Business. J. Occup. Environ. Med..

[38] Tenney L., Dexter L., Shapiro D.C., Dally M., Brown C.E., Schwatka N.V., Huebschmann A.G., McMillen J., Newman L.S. (2021). Impact of Advising on Total Worker Health Implementation. J. Occup. Environ. Med..

[39] Schwatka N.V., Dally M., Tenney L., Shore E., Brown C.E., Newman L.S. (2020). Total Worker Health Leadership and Business Strategies Are Related to Safety and Health Climates in Small Business. Int. J. Environ. Res. Public Health.

[40] Schwatka N.V., Dally M., Shore E., Dexter L., Tenney L., Brown C.E., Newman L.S. (2021). Profiles of total worker health^®^ in United States small businesses. BMC Public Health.

[41] Aarons G.A., Sklar M., Mustanski B., Benbow N., Brown C.H. (2017). “Scaling-out” evidence-based interventions to new populations or new health care delivery systems. Implement. Sci..

[42] Adams E.J., Chalkley A.E., Esliger D.W., Sherar L.B. (2017). Evaluation of the implementation of a whole-workplace walking programme using the RE-AIM framework. BMC Public Health.

[43] Moullin J.C., Dickson K.S., Stadnick N.A., Albers B., Nilsen P., Broder-Fingert S., Mukasa B., Aarons G.A. (2020). Ten recommendations for using implementation frameworks in research and practice. Implement. Sci. Commun..

[44] Waltz T.J., Powell B.J., Chinman M.J., Smith J.L., Matthieu M.M., Proctor E.K., Damschroder L.J., E Kirchner J. (2014). Expert recommendations for implementing change (ERIC): Protocol for a mixed methods study. Implement. Sci..

[45] (2019). Health Links. https://www.healthlinkscertified.org.

[46] Cunningham T.R., Sinclair R., Schulte P. (2014). Better understanding the small business construct to advance research on delivering workplace health and safety. Small Enterp. Res..

[47] Dearing J.W. (2009). Applying Diffusion of Innovation Theory to Intervention Development. Res. Soc. Work. Pr..

[48] Moullin J.C., Dickson K.S., Stadnick N.A., Rabin B., Aarons G.A. (2019). Systematic review of the Exploration, Preparation, Implementation, Sustainment (EPIS) framework. Implement. Sci..

[49] Baumann A.A., Cabassa L.J., Stirman S.W. (2017). Adaptation in dissemination and implementation science. Dissemination and Implementation Research in Health: Translating Science to Practice.

[50] Stirman S.W., Baumann A.A., Miller C.J. (2019). The FRAME: An expanded framework for reporting adaptations and modifications to evidence-based interventions. Implement. Sci..

[51] Kilbourne A., Neumann M., Pincus H.A., Bauer M., Stall R. (2007). Implementing evidence-based interventions in health care: Application of the replicating effective programs framework. Implement. Sci..

[52] Romppanen J.M. (2021). Segmentation, Targeting & Positioning (STP). https://www.researchgate.net/profile/Jan-Romppanen/publication/348923039_Segmentation_Targeting_Positioning_STP/links/607768762fb9097c0ce54a58/Segmentation-Targeting-Positioning-STP.pdf.

